# SIRT1 Negatively Regulates the Mammalian Target of Rapamycin

**DOI:** 10.1371/journal.pone.0009199

**Published:** 2010-02-15

**Authors:** Hiyaa Singhee Ghosh, Michael McBurney, Paul D. Robbins

**Affiliations:** 1 Department of Microbiology and Molecular Genetics, University of Pittsburgh School of Medicine, Pittsburgh, Pennsylvania, United States of America; 2 Ottawa Health Research Institute and Departments of Medicine and Biochemistry, Microbiology and Immunology, University of Ottawa, Ottawa, Canada; Roswell Park Cancer Institute, United States of America

## Abstract

The IGF/mTOR pathway, which is modulated by nutrients, growth factors, energy status and cellular stress regulates aging in various organisms. SIRT1 is a NAD+ dependent deacetylase that is known to regulate caloric restriction mediated longevity in model organisms, and has also been linked to the insulin/IGF signaling pathway. Here we investigated the potential regulation of mTOR signaling by SIRT1 in response to nutrients and cellular stress. We demonstrate that SIRT1 deficiency results in elevated mTOR signaling, which is not abolished by stress conditions. The SIRT1 activator resveratrol reduces, whereas SIRT1 inhibitor nicotinamide enhances mTOR activity in a SIRT1 dependent manner. Furthermore, we demonstrate that SIRT1 interacts with TSC2, a component of the mTOR inhibitory-complex upstream to mTORC1, and regulates mTOR signaling in a TSC2 dependent manner. These results demonstrate that SIRT1 negatively regulates mTOR signaling potentially through the TSC1/2 complex.

## Introduction

The NAD^+^ dependent deacetylase, SIRT1 (Sir2) has been shown to regulate a wide variety of cellular processes including aging and lifespan extension [1], [2], [3], [4]. Transgenic mice overexpressing SIRT1 have a beneficial Calorie Restriction (CR)-like phenotype, whereas downregulation of SIRT1 accelerates the aging phenotype in mice [5], [6]. Interestingly, SIRT1 orthologs are linked to the insulin/IGF signaling pathway in *C. elegans, drosophila* and mice through its ability to deacetylate the FOXO proteins [7], [8], [9]. For example, the longevity phenotypes in *C. elegans* are suppressed by mutations in daf-16, a forkhead family transcription factor, which is regulated by SIRT1 [10]. Notably, CR induces SIRT1 expression, which can be attenuated by IGF-1. Furthermore, treatment of cells with either insulin or IGF-1 lowers SIRT1 levels, suggesting an inverse relationship between SIRT1 and the insulin/IGF pathway [8]. However, the role of SIRT1 in CR induced longevity remains controversial because in yeast, the cyclic-AMP-dependent kinase (PKA) signaling pathway has been implicated in CR induced longevity, independent of Sir2 [11]. In addition, severe CR has been shown to involve the “target of rapamycin” (TOR) pathway for lifespan extension in yeast [12], [13].

The mammalian target of rapamycin (mTOR) is a serine/threonine protein kinase that regulates cell growth and proliferation by modulating protein synthesis and transcription. mTOR acts as nutrient, energy and redox sensor by integrating signals from multiple upstream signaling pathways, including insulin, growth factors (IGF1/2), and mitogens. The mTOR complex 1 (mTORC1) consists of mTOR, regulatory associated protein of mTOR (Raptor), LST8/G-protein β-subunit like protein (mLST8/GβL) and PRAS40. mTORC1 is stimulated by growth promoting conditions and inhibited by low nutrient levels, growth factor deprivation, reductive stress and the specific inhibitor of mTORC1, Rapamycin. Upstream to mTORC1 is the TSC1-TSC2 inhibitory complex, which functions as a GTPase activating protein (GAP) for the GTPase Rheb, an upstream activator of mTOR. The TSC1-TSC2 complex inactivates Rheb to inhibit mTOR signaling [14], [15]. Diverse growth and stress signals converge at the TSC1-TSC2 complex to regulate mTORC1 signaling.

The mTOR pathway has been implicated in longevity in model organisms such as yeast, worms and flies. Over-expression of the *Drosophila* homologs dTSC1 or dTSC2 or mutation in dTOR or its downstream target dS6K, leads to longevity phenotype in *Drosophila* [16]. In yeast, 6 out of 10 gene mutations that are known to increase replicative life span correspond to components of the TOR pathway including TOR and S6K1 (Sch9) [13]. Furthermore, TOR inhibition has been shown to extend lifespan in yeast by increasing Sir2p activity [17]. Resveratrol, a known activator of SIRT1, has been demonstrated to inhibit mTOR activity and cellular senescence [18], [19], [20]. In a recent extensive study, rapamycin, the inhibitor of mTOR, was shown to extend the median and maximal lifespan of mice [21].

The two best characterized substrates of mTORC1 are p70-S6 Kinase 1 (S6K1) and 4E-BP1, the eukaryotic initiation factor 4E (eIF4E) binding protein 1. Activation of mTOR results in phosphorylation of S6K1 and 4EBP1, which increases protein synthesis and ribosome biogenesis. Thus activation of mTOR results in an increase in cell size and mass [22].

Clearly mTOR and SIRT1 regulate many common effectors critical to the longevity signaling pathways in lower organisms and mice. However, no direct link has yet been established between these two important regulators. Here we investigated the potential functional interrelationship between these two proteins in regulating the stress response. Our results demonstrate that SIRT1 indeed regulates mTOR signaling, potentially through TSC2.

## Results

### SIRT1 Regulates mTOR Signaling in Human and Mouse Cells

**Figure 1:**
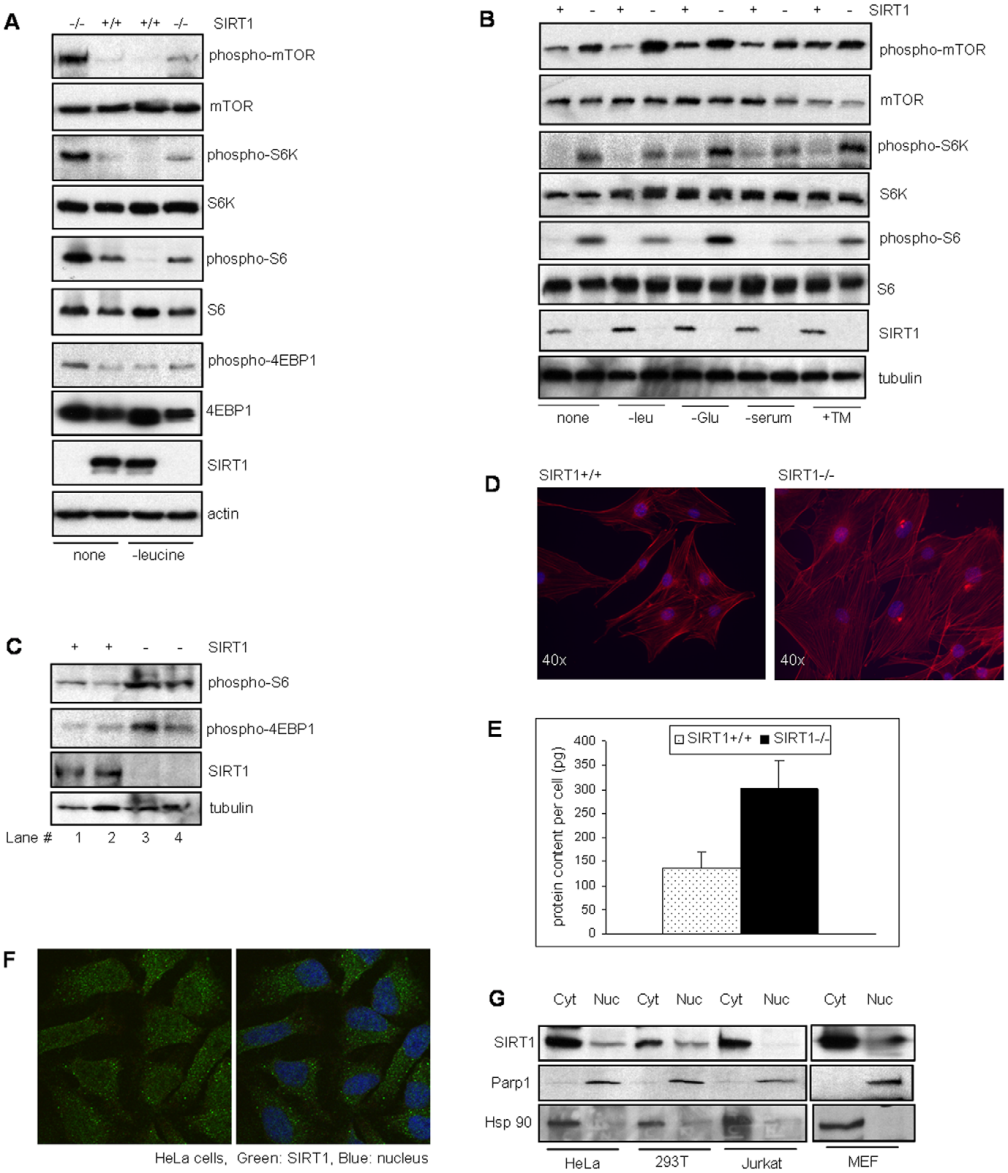
mTOR activity in SIRT1 deficient mouse and human cells. (A) Western blot analysis for phosphorylation of the various mTOR target protein substrates from mouse embryonic fibroblasts (MEFs) that were either un-treated or leucine starved. (B) Western blot analysis of extracts from HeLa cells depleted for SIRT1 after stress treatments as indicated. (C) Western blot analysis of muscle extracts from WT or SIRT1 knock-out mice. Lane 1 and 2 are tissues from two WT mice, and Lane 3 and 4 are tissues from two SIRT1 null mice. (D) F-actin (red) staining of SIRT1 null and wild-type (WT) MEFs. (E) Protein content of SIRT1+/+ and SIRT1−/− MEFs. (F) Immunofluroscence of HeLa cells using SIRT1 monoclonal antibody. (G) Subcellular fractionation of HeLa, 293T, Jurkat cells and MEFs followed by Western blotting for SIRT1. Anti-Parp1 and Hsp90 shows the nuclear and cytoplasmic fractionation. -leu: leucine deprived, -glc: glucose deprived, -ser: serum deprived, +TM: tunicamycin.

We investigated the activity of mTOR pathway in SIRT1 deficient mouse embryonic fibroblasts (MEFs) by analyzing the phosphorylation of mTOR and its substrates S6K1 and 4EBP1. Phosphorylation of S6, the downstream target of S6K1 was also analyzed. As shown in Figure 1A, absence of SIRT1 resulted in higher phosphorylation of mTOR, S6K1, 4EBP1 and S6, suggesting a role for SIRT1 in mTORC1 regulation. Since mTOR pathway is responsive to nutrient and cellular stress and is downregulated in response to stress signals, we examined if stress induced by amino acid (leucine) starvation inhibited the upregulated mTOR signaling in SIRT1 deficient cells. We observed that in contrast to WT cells, the upregulated mTOR activity in SIRT1 null cells was not fully abrogated even under leucine starvation.

To verify these results in human cells, HeLa cells were depleted for SIRT1 using stable retroviral delivery of an shRNAi specific for SIRT1 [23]. The SIRT1 depleted and matched-control HeLa cells were then treated with a number of different stress conditions and phosphorylation of mTOR, S6K1 and S6 examined. Consistent with our data from murine cells, the SIRT1-depleted HeLa cells showed higher mTOR signaling regardless of the stress conditions (Figure 1B). To further confirm the role of SIRT1 in regulating mTOR pathway in animals, we analyzed the mTOR pathway in SIRT1^−/−^ and WT mouse tissue. As indicated by higher levels of phospho-S6 and phospho-4EBP1 (Figure 1C), tissues from SIRT1 knock-out mice showed elevated mTOR activity.

Consistent with a role for mTOR in regulating cell size by modulating protein synthesis, we also observed a larger morphology for the SIRT1 deficient MEFs compared to WT MEFs (Figure 1D). Furthermore, on a single cell level, the SIRT1 deficient cells showed a higher protein content per cell when compared to wild-type MEFs (Figure 1E). SIRT1 is predominantly known as a nuclear protein, although several recent reports suggest the cytoplasmic presence of SIRT1 [24], [25]. Since mTOR signaling is a cytoplasmic process, we examined the intracellular localization of SIRT1 by immunofluroscence of HeLa cells and subcellular fractionation of several human cell lines and mouse embryonic fibroblasts. Our results demonstrate that SIRT1 is present in the cytoplasm of HeLa, 293T, Jurkat cells and mouse embryonic fibroblasts, consistent with a cytoplasmic role for SIRT1 (Figure 1F and G).

### Deacetylase Activity of SIRT1 Plays a Role in mTOR Signaling

**Figure 2:**
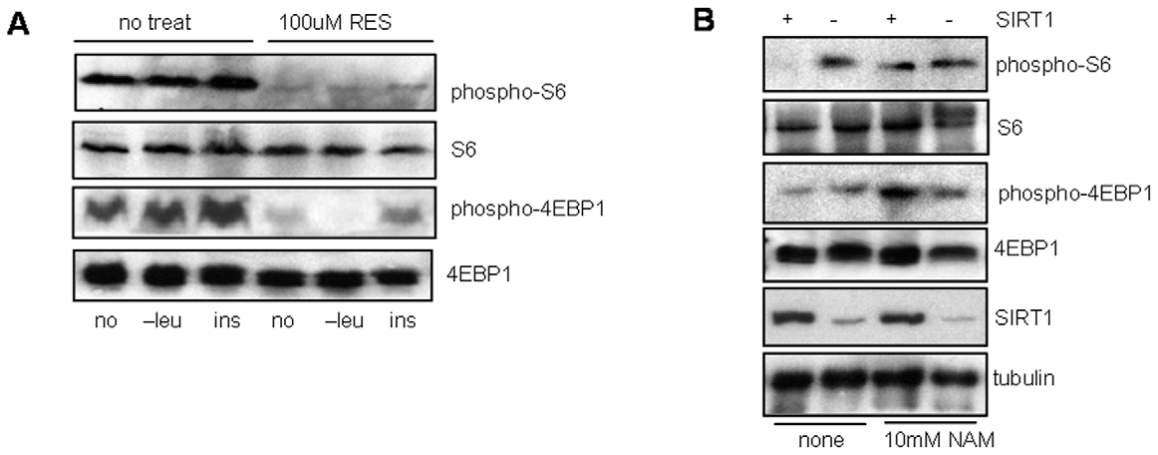
mTOR signaling in response to SIRT1 activator (resveratrol) and inhibitor (nicotinamide). (A) HeLa cells were either mock-treated (vehicle alone) or treated with –leucine media or 200nM insulin, with or without 50 µM resveratrol as indicated. (B) SIRT1-depleted and control HeLa cells were either mock-treated or treated with 10mM nicotinamide.

SIRT1 is a NAD+ dependent deacetylase whose catalytic activity is important for most of its known functions. To determine if the catalytic activity of SIRT1 is important for regulation of mTOR signaling, HeLa cells were treated with the SIRT1 activator resveratrol (RES), under stress (-leucine) or growth (insulin) conditions and mTOR signaling measured by examining phosphorylation levels of S6 and 4EBP1 (Figure 2A). Resveratrol suppressed mTOR signaling regardless of stress or growth conditions, suggesting that inducing the catalytic activity of SIRT1 negatively regulates mTOR signaling. To further confirm this observation, matched-control and SIRT1-depleted HeLa cells were treated with the SIRT1 inhibitor nicotinamide (NAM). Consistent with the resveratrol results, the NAM treated control cells showed upregulation of S6 and 4EBP1 phophorylation (Figure 2B). This result further demonstrates that the catalytic activity of SIRT1 is important for mTOR regulation and inhibition of the catalytic activity results in elevated mTOR signaling in normal cells.

### Rapamycin Inhibits Upregulated mTOR in SIRT1 Deficient Cells

**Figure 3:**
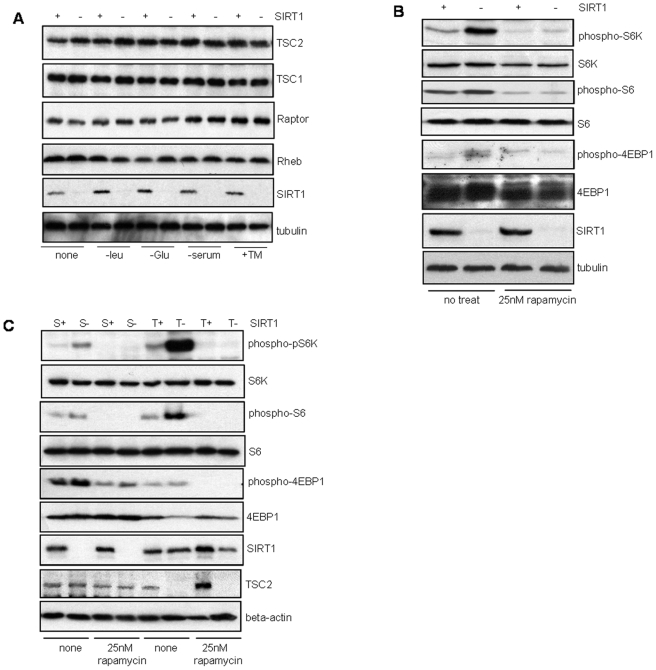
Expression and signaling in mTOR pathway in response to stress stimuli and rapamycin. (A) Western blot analysis of extracts from matched-control and SIRT1 depleted HeLa cells after stress treatments as indicated. (B) SIRT1-depleted and matched-control HeLa cells, and (C) Wild-type, SIRT1 null and TSC2 null MEFs were either mock-treated (vehicle alone) or treated with 25nM rapamycin for 1 hr followed by Western blot analysis for mTOR activity. S+: SIRT1+/+, S-: SIRT1−/−, T+: TSC2+/+, T-: TSC2−/−.

To determine if SIRT1 modulates the expression of the critical proteins involved in regulation of the mTOR pathway, protein expression analysis of TSC1, TSC2, Raptor and Rheb in SIRT1 depleted cells under various stress conditions was performed. As shown in figure 3A, SIRT1 deficiency does not affect the expression of the mTOR regulatory proteins.

To determine if SIRT1 regulates the mTOR signaling pathway upstream or downstream from mTORC1, we examined whether rapamycin, a specific mTOR inhibitor that inhibits mTOR complex 1 (mTORC1) activity, could inhibit the upregulated mTOR activity in the SIRT1 depleted cells. Treatment of the matched-control or SIRT1 depleted HeLa cells with rapamycin significantly abrogated the phophorylation of S6K1, S6 and 4EBP1 (Figure 3B), suggesting that SIRT1 regulates mTOR upstream of the mTORC1.

mTOR is downregulated by the upstream TSC1/TSC2 complex. TSC2 null cells show upregulated mTOR signaling whereas over-expression of TSC1 and TSC2 result in mTOR inhibition [26], [27]. Thus we examined if rapamycin could inhibit upregulated mTOR signaling in SIRT1 deficient MEFs compared to TSC2 deficient MEFs. Treatment of both TSC2−/− and SIRT1−/− MEFs with rapamycin resulted in abrogation of the elevated mTOR activity, demonstrating that SIRT1 null MEFs are similarly sensitive to rapamycin as the TSC2 null MEFs (Figure 3C). Interestingly, in contrast with the SIRT1 null MEFs, the TSC2 null cells did not show a significant size difference from the TSC2+/+ MEFs (Figure S1).

### SIRT1 Inhibits mTOR Signaling through TSC2

**Figure 4:**
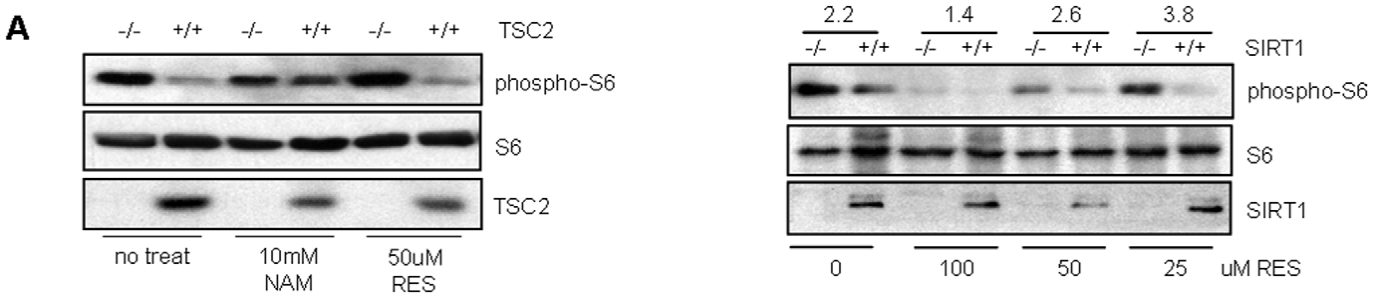
Resveratrol's effect on mTOR activity in TSC2 and SIRT1 null cells. (A) Wild-type and TSC2 null MEFs were either mock treated or treated with 10 mM nicotinamide or 50 µM resveratrol as indicated. (B) Wild-type and SIRT1 null MEFs were mock-treated or treated with 25, 50 or 100 µM resveratrol as indicated. The ratio of band intensities between the SIRT1 null and corresponding WT MEFs for each group was calculated after normalizing the phospho-protein signals with the total protein signals. The calculations were done using ImageJ software.

Since the above results suggested that SIRT1 acts upstream of mTORC1 to downregulate mTOR signaling similar to TSC2, we investigated if the SIRT1-mediated down-regulation of mTOR signaling was TSC2 dependent. We treated WT and TSC2 null MEFs with the SIRT1 activator resveratrol (RES), and SIRT1 inhibitor nicotinamide (NAM). As expected, only the TSC2 null MEFs showed an increased S6 phosphorylation. In contrast, NAM treatment induced S6 phosphorylation in WT MEFs (Figure 4A), suggesting a potential role for SIRT1 in TSC2-mediated inhibition of mTORC1 activity. Interestingly, the SIRT1 activator, RES, could not inhibit S6 phosphorylation in absence of TSC2, indicating that SIRT1 may be dependent on TSC2 for inhibiting mTORC1 activity. In addition, no further decrease in S6 phosphorylation in response to RES treatment was observed in WT cells, possibly due to the lower basal levels of S6 phosphorylation in WT cells which are both TSC2 and SIRT1 positive.

Resveratrol has been shown to activate the AMP-activated kinase (AMPK) pathway independent of SIRT1 [28], [29]. AMPK, a cellular energy sensor, also acts as a negative regulator of mTOR pathway by activating TSC2 in response to energy stress. However, consistent with another published report [30] we could not detect any measurable difference in AMPK activity in the absence of SIRT1 (Figure S2), but instead detected significant differences in mTOR activity.

To confirm that the observed effects of resveratrol were indeed mediated through SIRT1, we investigated the effect of resveratrol on WT versus SIRT1 null MEFs. Resveratrol treatment strongly inhibited S6 phosphorylation in WT MEFs, but with reduced efficacy in SIRT1 null MEFs, suggesting that the effect of resveratrol on mTOR signaling is mediated partly through SIRT1 (Figure 4B). We observed that the effect of resveratrol on mTOR signaling was dose-dependent with lower concentrations (25 µM) showing inhibition only in SIRT1-positive cells, while higher concentrations (100 µM) inhibited mTOR both in WT as well as in the SIRT1-deficient cells. This result suggests that resveratrol regulates mTOR signaling through both SIRT1-dependent and -independent pathways, consistent with reports showing SIRT1 independent effects of resveratrol.

### SIRT1 Interacts with TSC2

**Figure 5:**
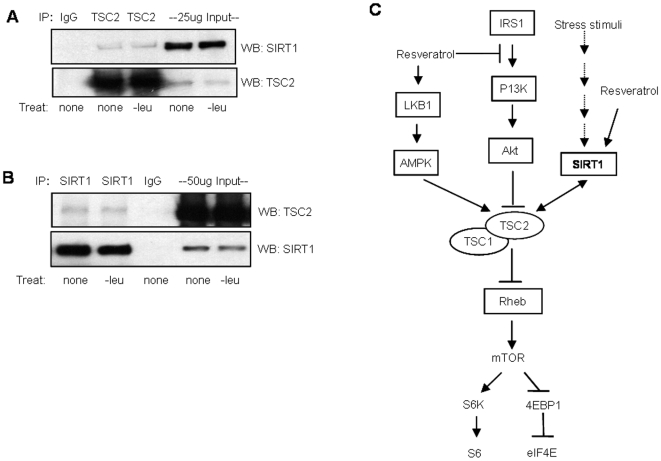
SIRT1 associates with TSC2. (A) Immunoprecipitation of TSC2 from HeLa cells that were either un-treated or leucine deprived, followed by Western blot analysis for SIRT1. (B) Immunoprecipitation of SIRT1 from whole cell extracts of cells that were either mock-treated or treated with leucine deprived media followed by Western blot analysis of the immunoprecipitated extracts for TSC2. (E) Schematic of SIRT1 and resveratrol mediated regulation of the mTOR complex-1, conferred through the TSC1/TSC2 complex. SIRT1 associates with TSC2 and downregulates mTOR signaling in response to stress stimuli. Resveratrol regulates mTOR signaling through both SIRT1-dependent and -independent pathways.

Given that the effects of RES and NAM on mTOR regulation are both SIRT1- and TSC2-dependent, we investigated if these two proteins interact *in vivo*. Immunoprecipitation of endogenous TSC2 resulted in coimmunoprecipitation of endogenous SIRT1 (Figure 5A) and conversely, TSC2 was coimmunoprecipitated with SIRT1 (Figure 5B) with or without stress condition (-leucine), indicating that SIRT1 associates with TSC2. We further examined if TSC2 is an acetylated protein. Although we could not detect acetylation of TSC2 by immunoblotting and by mass-spec analysis (data not shown), it is still possible that TSC2 or another protein in the complex is acetylated in response to stress or growth conditions and can be regulated by the deacetylase activity of SIRT1.

## Discussion

Both SIRT1 and mTOR have been linked to age-associated diseases with SIRT1 activation having a protective effect, whereas inhibition of mTOR conferring a beneficial effect. For example, SIRT1 activation confers a therapeutic effect in type 2 diabetes, obesity and neurodegenerative diseases such as Alzheimer's and amyotrophic lateral sclerosis, whereas inhibition of mTOR is protective against cardiovascular and neurological diseases, diet-induced obesity and cancer [31], [32], [33], [34], [35], [36], [37], [38]. Autophagy, a mechanism important in regulating stress response and aging is negatively regulated by mTOR [39], [40], whereas SIRT1 has been reported to activate autophagy by deacetylating several essential components of the autophagy machinery [41].

The inverse relationship between the roles of SIRT1 and mTOR in aging-associated diseases and lifespan extension suggests a functional interrelationship between these two proteins. Our results demonstrate that SIRT1 and mTOR signaling pathways are indeed interconnected in a way that promotes stress sensing pro-survival signals, where the regulation of mTOR is mediated potentially through an interaction of SIRT1 with the TSC1-TSC2 complex.

Stress conditions downregulate mTOR signaling thereby reducing protein synthesis and cell growth. We found that this mechanism is deregulated in the absence of SIRT1 in mouse and human cells. SIRT1 deficiency caused upregulation of mTOR signaling which could not be abolished even under cellular stress caused by leucine starvation and other stress inducible stimuli. Interestingly, SIRT1 has been suggested as a nutrient-sensitive growth suppressor gene [42]. Although it was proposed that SIRT1 functions through regulation of telomerase activity, our results suggest that SIRT1 functions as a nutrient-responsive growth suppressor also by regulating mTOR signaling.

SIRT1 has been shown to regulate many metabolic and stress responsive pathways through the regulation of gene expression of critical components. We observed that for mTOR regulation, SIRT1 does not seem to function through regulating expression of mTOR signaling proteins, instead SIRT1 potentially regulates mTOR through an upstream inhibitory complex. Using SIRT1 deficient and TSC2 deficient cells, we observed that SIRT1's inhibitory effect on mTOR was similar to that of the mTOR inhibitory protein TSC2. Further analysis using SIRT1 activator and inhibitor indicated that the mTOR inhibitory effect of SIRT1 was at least partially dependent on TSC2. Resveratrol has been reported to affect insulin signaling through SIRT1 independent pathways. Consistent with these reports, our data demonstrated that at lower doses, resvetratrol regulated the mTOR pathway in a SIRT1 dependent manner. However, at higher doses, reveratrol likely activated SIRT1 independent pathways in parallel, to inhibit mTOR activity.

Based on our data, we propose a model where negative regulation of mTOR signaling by SIRT1 is mediated through its association with TSC2 (Figure 5C). The TSC1-TSC2 complex is the most prominent upstream inhibitor of mTOR signaling, integrating several upstream signals such as growth factor, energy, stress and possibly amino acids. In response to specific stimuli, such as hormones, low energy, low nutrient or hypoxia, specific kinases and regulatory proteins activate or inhibit the TSC2 protein of the TSC1-TSC2 complex, thereby regulating mTOR signaling. By acting through the main mTOR inhibitory complex (TSC1/TSC2 complex), SIRT1 potentially responds to more than one form of stress or growth signal to regulate mTOR signaling.

Importantly, our results demonstrating a role for SIRT1 in mTOR signaling is the first evidence for SIRT1 to directly modulate translation-regulation. Previously, SIRT1's role in regulating cellular stress response was shown to involve various transcription factors such as NF-κB, p53 and the FOXO proteins, and other non-transcription factor proteins such as Ku70 and ATG [8], [41]. Consistent with a role for SIRT1 in stress induced translation regulation, we also have demonstrated that SIRT1 interacts with eIF-2alpha, a translation initiation factor, to regulate stress induced translation control (Ghosh et al).

Future studies are needed for insight into the exact mechanism of SIRT1's regulation on the TSC1/TSC2 complex or other potential upstream regulators of mTORC1. Since both SIRT1 and mTOR affect cellular pathways critical in stress response and aging, the regulatory inter-relationship between these proteins will prove to be helpful for designing effective therapeutic strategies for age-associated diseases.

## Materials and Methods

### Cell Lines and Mouse Tissues

HeLa and 293 cells (obtained from the American Type Culture Collection) were maintained under standard cell culture conditions. WT and SIRT1 null mouse embryonic fibroblasts [43] were maintained in 15% oxygen under normal culture condition. HeLa cell line depleted for SIRT1 was generated by stable retroviral transfection of shRNAi directed against the human SIRT1 gene as described [23]. The matched (negative) control cells were transfected with a negative control non-targeting shRNAi (BD Biosciences). TSC2+/+ and TSC2-/- MEFs were a kind gift from Dr. David Kwiatkowski (Harvard Medical School).

### Cell Size Determination

For cell size determination, cells were stained for F-actin using rhodamine labeled phalloidin stain. The nucleus was stained with DAPI. Actin staining was visualized by fluorescent microscopy at 40X magnification.

### Western Blot Analysis

Whole cell extracts of WT and SIRT1 null MEFs and HeLa cells were made using NP-40 lysis buffer with standard protease inhibitors (Sigma Aldrich protease inhibitor cocktail) followed by western blot analysis for phosphorylation of the mTOR substrates using phospho-protein-antibody (Cell Signaling Technology) for various mTOR substrate proteins. All treatments; -leucine, -glucose, -serum, tunicamycin (2 µg/ml) were given for 1hr before making extracts for western blot analysis.

### Subcellular Localization of SIRT1

HeLa cells were immunostained for SIRT1 using SIRT1 monoclonal antibody (Upstate). The nucleus was stained with DAPI. Localization of SIRT1 was measured by fluorescent microcopy at 40X. Subcellular fractionation of HeLa, 293T, Jurkat cells and MEFs was performed using Pierce fractionation kit.

### Co-Immunoprecipitation

For co-immunoprecipitation, whole cell extracts were made in NP-40 lysis buffer with standard protease inhibitors. All treatments were given for 1hr prior to making the cell lysates. Approximately 2-3 million cells were used. The immunoprecipitation was performed on 500 µg protein extract and 5% (25ug) of the immunoprecipitates were used as the input. SIRT1 antibody from upstate and TSC2 antibody from cell signaling technology was used for western blotting.

## Supporting Information

Figure S1 F-actin staining of TSC2+/+ and TSC2 −/− MEFs: Actin was stained using rhodamine-phalloidin stain. Nucleus was stained with DAPI. Red: F-actin, Blue: nucleus.
(0.51 MB TIF)

Figure S2 AMPK activity in Control and SIRT1-RNAi HeLa cells: cell lysates from untreated or leucine starved cells were analysed by Western blot analysis using phospho-AMPK antibody (Cell Signaling technology). Tubulin is shown as loading control.
(0.08 MB TIF)
