# Supplementary figures and images for: SIRT1 Negatively Regulates the Mammalian Target of Rapamycin
Source: PLoS One. 2010 Feb 15;5(2):e9199. doi: 10.1371/journal.pone.0009199 (PMC2821410; doi:10.1371/journal.pone.0009199)

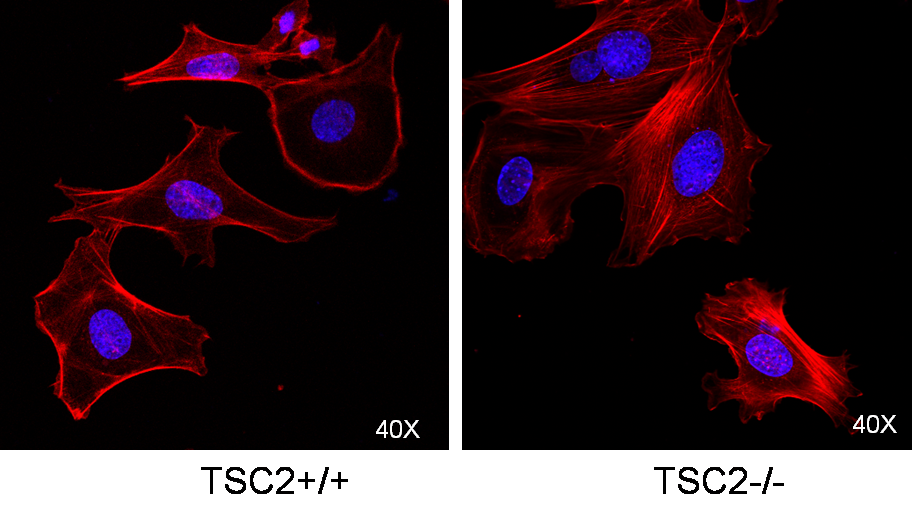

Supplement: Figure S1 — F-actin staining of TSC2+/+ and TSC2 −/− MEFs: Actin was stained using rhodamine-phalloidin stain. Nucleus was stained with DAPI. Red: F-actin, Blue: nucleus. (0.51 MB TIF) [file pone.0009199.s001.tif]

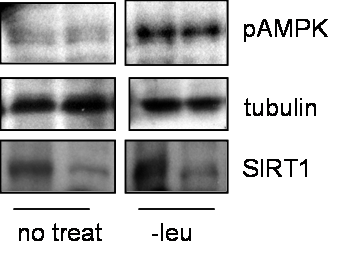

Supplement: Figure S2 — AMPK activity in Control and SIRT1-RNAi HeLa cells: cell lysates from untreated or leucine starved cells were analysed by Western blot analysis using phospho-AMPK antibody (Cell Signaling technology). Tubulin is shown as loading control. (0.08 MB TIF) [file pone.0009199.s002.tif]
